# Site Selection for Underground Hydrogen Storage in Porous Media: Critical Review and Outlook

**DOI:** 10.1021/acs.energyfuels.5c03665

**Published:** 2025-09-12

**Authors:** Willemijn A. van Rooijen, Hadi Hajibeygi

**Affiliations:** Reservoir Engineering, Geoscience and Engineering Department, Faculty of Civil Engineering and Geosciences, 2860 Delft University of Technology, Stevinweg 1, 2628CN Delft, The Netherlands

## Abstract

Underground hydrogen storage in porous media is promising for large-scale energy storage. However, its technical and financial effectiveness is heavily dependent on a reliable site selection strategy. In this review, we critically assess the available literature across disciplines to identify the most influential criteria for reliable site selection. Drawing from this evaluation, we propose a systematic, multidisciplinary framework for early stage reservoir screening, integrating key criteria from reservoir performance, geomechanics and containment, location and techno-economics, and biogeochemistry. Our framework allows for rapid identification and ranking of the most suitable reservoirs by proposing 11 elimination criteria and 15 screening criteria. The presented framework consists of practically applicable and scientifically grounded criteria to support consistent, early stage decisions based on readily available data while allowing for detailed site-specific analysis in later project development phases. By unifying diverse disciplinary insights into a structured methodology, this study contributes to more informed, inclusive, and effective site selection.

## Graphical abstract

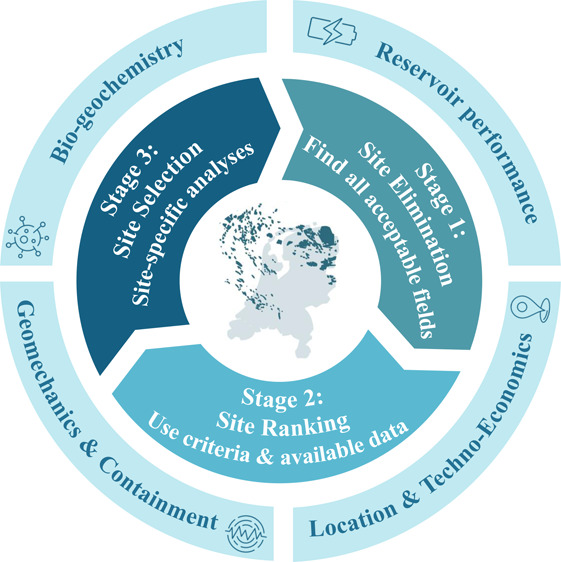

## 1 Introduction

As the global energy transition accelerates, hydrogen is considered as an important energy carrier, in particular for long-term energy storage, due to its high gravimetric energy density and clean combustion products.^1–4^ Underground Hydrogen Storage (UHS) in geological formations, such as salt caverns, depleted reservoirs, and aquifers, is promising for large-scale, long-term energy storage to balance supply and demand fluctuations.^5–8^ Depleted gas reservoirs offer significant potential due to their high capacity.^9–11^ However, selecting a suitable reservoir is a crucial step in enabling a safe and efficient storage implementation project.^12^ This selection process must account for a range of factors, which are multidisciplinary by nature, including reservoir performance (e.g., injectivity and deliverability), geomechanical stability, containment integrity, biogeochemical interactions, and techno-economic viability. A comprehensive site ranking and selection framework, which is developed based on a critical and inclusive assessment of these dimensions, is essential to mitigate risks and maximize the operational efficiency.

While Underground Gas Storage (UGS) has been widely utilized for several decades, underground hydrogen storage remains a relatively new technology. Despite the long history of gas storage, publicly available research on the site selection criteria for underground gas storage fields remains limited.^13–15^ In contrast, site selection for CO_2_ storage has been quite extensively investigated, with numerous studies providing comprehensive screening criteria and assessment methodologies.^15–24^ Site selection of Underground Hydrogen Storage has been gaining more attention in recent years. Numerous studies describe workflows for selecting suitable UHS fields.^12,25–35^

Many of these studies employ multicriterion decision-making (MCDM) techniques, such as the Analytic Hierarchy Process, to evaluate a range of site selection criteria and their relative importance. Expert judgment plays a crucial role in this context, especially given the diverse and often noncomparable nature of the factors involved. However, to ensure that site selection decisions are grounded in robust evidence, it is important to complement expert-based methods with quantitative techniques, such as sensitivity analysis through reservoir modeling. Sensitivity analysis systematically evaluates how variations in key geological and operational parameters impact storage performance, leading to a more reliable and evidence-based site selection. Despite its value, only a few studies have applied this approach for site selection.^26,31,36^ Okoroafor et al.^26^ identified critical parameters for hydrogen storage in depleted gas fields, while Sekar et al.^31^ extended this to saline aquifers, proposing elimination and ranking criteria. Chen et al.^36^ developed a machine learning-based reduced-order model trained on high-fidelity simulations to perform sensitivity analysis and identify optimal saline aquifers in the U.S. Similarly, Malki et al.^32^ introduced OPERATE-H2, a tool that integrates reduced-order models and sensitivity analyses to assess hydrogen storage performance and support site selection based on geologic and operational factors. Although Wang et al.^11^ and Mwakipunda et al.^37^ addressed site selection as part of a broader review, their treatment of this topic is limited in detail due to the wider scope of their studies.

In addition to the studies explicitly addressing site selection, numerous other investigations offer valuable insights by focusing on specific aspects of UHS. These studies often employ sensitivity analyses or explore individual parameters or aspects such as geological heterogeneity,^38^ microbiological activity,^39,40^ or residual trapping. By concentrating on isolated factors, they provide a more detailed understanding of the underlying physical and operational processes, thereby contributing critical knowledge that complements broader site selection frameworks. Such focused analyses enhance the overall understanding of the UHS system behavior and can inform the development of more robust science-based selection criteria.

Indeed, as more pilot projects for UHS are initiated worldwide, a comprehensive and scientifically grounded approach to site selection is becoming increasingly important. However, no comprehensive review currently exists that specifically focuses on UHS site selection, the very first crucial step in making any project successful. And, to the best of the authors’ knowledge, a scientifically grounded framework that integrates insights from all relevant disciplines into quantifiable and practically applicable criteria for site selection is also missing in the literature. This review and outlook study addresses such a significant knowledge gap by presenting a systematic, multidisciplinary approach for reliable site selection for UHS in porous media. Here, we focus on the most influential and practically applicable criteria to enable rapid and effective field elimination and ranking among the many available options. The framework draws directly from established findings in the literature, including results from sensitivity analyses and domain-specific studies, to ensure both scientific rigor and practical applicability.

We begin by reviewing and evaluating existing studies on site selection for UHS, identifying current practices and their limitations (Section 3). To provide context, we then compare site selection approaches across UHS, underground gas storage (UGS), and carbon capture and storage (CCS), highlighting the differences in site selection methodologies across these applications (Section 3). Building on these insights, we introduce a framework setup specifically developed for UHS (Section 4). Its structure is organized around four main categories: Reservoir Performance, Biogeochemistry, Geomechanical Risks and Containment, and Location and Techno-Economics. Each category is analyzed to identify the most critical factors influencing site suitability (Sections 5–8). These elements are then integrated into a comprehensive framework (Section 9), followed by a discussion of key challenges and perspectives (Section 10) and a conclusion (Section 11).

## 2 Site Selection Strategies: Overview and Assessment

Site selection for Underground Hydrogen Storage has received increasing attention in recent literature.^12,25–31,33–36,41–45^ Numerous studies have applied site selection methods to specific regions, including Japan, Germany, the UK, USA, and Poland.^12,28,30,31,33,36,44,45^

Site selection is typically based on predefined criteria, which play a crucial role in determining suitable locations. Some studies propose new, UHS application specific, site selection criteria,^26,31,41,44^ while others derive their criteria from the existing literature,^25,28,30,35,42,44,45^ sensitivity analyses via reservoir model simulations,^26,31,36^ or even expert judgments. However, in some cases, the rationale behind the employed criteria is not explicitly stated, nor justified.^29,41,43^ Notably, very few studies provide a solid scientific basis for the proposed site selection criteria.

Table 1 provides an overview of the site selection criteria identified in UHS site selection studies. We have grouped the criteria into four categories; however, it is important to note that some criteria influence multiple categories. Most existing studies place a strong emphasis on reservoir performance, while biogeochemistry and geomechanical aspects receive comparatively less attention, and techno-economic factors, despite being very important in realization of the projects, are the least frequently addressed topics. Striking a balance between these areas remains a key challenge, as many studies consider only a subset of the criteria, resulting in an incomplete perspective on site suitability. Table 1 shows a wide range of criteria, and while some of the criteria, such as depth, permeability, and porosity, are relatively intuitive to evaluate, many others, e.g., cushion gas requirement, initial capital investment, and local culture embedding, most likely require additional analysis, which can become very time-consuming and challenging, especially as they can be beyond the technical aspects (including social sciences).

**Table 1:** Overview of All Criteria Used in Literature Site Selection Studies

reservoir performance	location and techno-economics
• pore volume/Storage capacity^12,25–28,34,35,41^	• labour^25^
• depth^12,25–29,31,35,41,42,44^	• proximity to suppliers and infrastructure^25,28,34,35^
• pressure^26,27,31,35,41,42^	• infrastructure availability^25,34^
• permeability^25–29,31,35,41,42,44^	• storage cost^25,35^
• porosity^25–29,31,35,41,42,44^	• initial investment^25,35^
• permeability anisotropy^26,27^	• regional risks^25,35^
• permeability heterogeneity^26,27^	• legal restrictions^25,34,41^
• closure/spill point^29,41^	• social acceptance^25,34,41^
• reservoir dip^26,27,31^	• job creation^25^
• reservoir structure^25,28,31,41,42^	• local culture^25^
• geothermal gradient^26,31^	• cushion gas requirement^27,28^
• stage of exploration^12^	• facilities, pipelines, ports^27^
• reservoir type (gas/oil/aquifer)^12,26,34,41^	• sensitive areas, environment^27^
• area^25,29,41^	• offshore/onshore^28,34^
• thickness^25,26,29,31,35,41,42,44^	• spatial planning^29^
• vertical closure^42^	
• flow capacity^28,34,44^	
• pressure buildup^44^	
• column height^44^	
• (vertical) net gross^28,29^	
• max. H_2_ well deliverability rate^28^	

geomechanical risks and containment	bio-geochemistry
• overburden rock lithology^12,27,42^	• rock types and mineralogy^27,31,34,41,42,44^
• faults in proximity, compartmentalization^26,27,29,31,34,41^	• knowledge of depositional environment^41^
• earthquakes/seismicity/tectonic activity^12,25–27,29,31,34,41^	• temperature^31,34,41,44^
• cap rock permeability^25,34^	• pressure^26,27,31,41^
• cap rock thickness^26,27,29,31,42^	• fluid characteristics, salinity, pH^27,31,34,41,44^
• secondary confining units^26,31^	• presence of microorganisms^27^
• overlying aquifers^34,41^	
• faults in overburden^29,34^	
• seal lithology^29^	
• subsidence^41^	
• proven seal^34,41^	
• well density^34^	

Moreover, the way these criteria are applied in the site selection process in the literature varies significantly. In our view, both screening and ranking are essential steps in the site selection process. However, screening, the elimination of fields that do not meet essential criteria, is not always conducted in the literature, which may lead to the selection of fields that lack essential characteristics. A structured and systematic approach that incorporates both phases of screening and ranking is necessary for reliable site selection.

An effective way to prioritize and weigh the various criteria is by using Multi-Criteria Decision-Making (MCDM) tools.^12,25,28–30,35,43^ These tools help to reduce subjectivity in site selection by ensuring a structured decision-making process and allowing for the comparison of criteria of different natures. Commonly used MCDM methods include the Analytic Hierarchy Process (AHP), the fuzzy Delphi method, and the Preference Ranking Organization Method for Enrichment Evaluations (PROMETHEE). While MCDM methods provide valuable insights, expert judgment remains integral to the process. This is particularly important given the wide range of factors involved, which are often difficult to directly compare. However, expert assessments can be inherently subjective, and the complexity of some MCDM methods can make the process of selecting a field more time-demanding.

Existing methods offer valuable insights, but a more systematic approach that integrates all of the important aspects is necessary for optimal UHS site selection. Additionally, scientific validation and quantification of the site selection criteria are imperative. Lastly, we focus on providing only the most influential site selection criteria to keep the method fast, efficient, and understandable for a wider public.

In our view, the following conditions are crucial for a robust site selection method: (a) quantified site selection criteria based on scientific research focusing only on the most influential criteria, (b) a structured approach incorporating both screening and ranking phases, and (c) consideration of all important aspects including: reservoir performance, biogeochemistry, geomechanical risks and containment, and location and techno-economics.

## 3 Site Selection of UHS vs UGS and CCS

Although large-scale Underground Hydrogen Storage (UHS) is not yet widely implemented, the established practices of Underground Gas Storage (UGS) and Carbon Capture and Storage (CCS) offer relevant insights.^4^ For example, decades of storage of methane in the subsurface have equipped us with a good understanding of risks associated with cyclic loading. However, key differences in gas properties must be considered when selecting suitable UHS sites.

Hydrogen differs significantly from methane (CH_4_) and carbon dioxide (CO_2_) in the density and viscosity. Under the conditions of 40 °C and 110 bar, as shown in Table 2, H_2_ is approximately 10 times lighter than CH_4_ and 45 times lighter than CO_2_, with viscosity also being lower by factors of 1.5 and 6, respectively. These differences strongly affect the dimensionless gravity number (Γ), which characterizes the balance between buoyancy and viscous forces,^46,47^ i.e.,

$$\Gamma =\frac{2\pi \Delta \rho gkH^{2}}{Q\mu_{\mathrm{brine}}}$$ 1

Here, Δρ is the density difference between the gas and the brine, *g* is the gravitational acceleration, *k* is the permeability, *H* is the reservoir thickness, *Q* is the injection rate, and μ_brine_ is the dynamic viscosity of the injected brine. As indicated in Table 2, Γ is about 7% higher for UHS compared to UGS and roughly 3 times higher than for CCS, signifying stronger buoyancy-driven flow in UHS, especially in comparison to CCS.^48^ This effect can be used to keep H_2_ segregated from other gases, but it also causes a risk for gravity overriding which should be taken into account.^49–51^ While hydrogen’s low viscosity and therefore high mobility enhance injectivity, it also increases the risk of unstable (fingering) displacement during injection, which can be assessed using the mobility ratio (*M*),^52^ defined as

$$M=\frac{k_{r\;\mathrm{gas}}/\mu_{\mathrm{gas}}}{k_{r\;\mathrm{brine}}/\mu_{\mathrm{brine}}}$$ 2

where *k*_r gas_ and *k*_r brine_ are the relative permeabilities of the gas and the brine, and μ_gas_ and μ_brine_ are the dynamic viscosities of the gas and the brine. A higher mobility ratio implies unfavorable displacement of brine by the mobile gas. As shown in Table 2, UHS exhibits a higher mobility ratio compared to UGS and CCS (assuming equal relative permeability values for the three gases), which indeed increases the likelihood of viscous fingering.^49–51^

**Table 2:** Comparison of Fluid Properties and Dimensionless Numbers for Underground Storage Systems^a^

parameter	H_2_/UHS	CH_4_/UGS	CO_2_/CCS
Thermophysical Properties of the Gas			
density [kg/m^3^]	8.0	78	680
viscosity [μPa s]	9.3	14	54
interfacial tension with brine [mN/m]	66	58	39
Dimensionless Numbers (Normalized to UHS)			
mobility ratio	1.0	0.67	0.18
gravity number (Γ)	1.0	0.93	0.36
Bond number (*N*_B_)	1.0	0.95	0.90

a Thermophysical properties are specific to the gas under reservoir conditions (40 °C and 110 bar). Dimensionless numbers are system-scaled and normalized relative to the underground hydrogen storage. A brine density of 1066 kg/m^3^ ^58^ and viscosity of 788 μPa s^59^ were assumed to calculate the dimensionless numbers.^60–63^

Potential leakage through caprock is often raised due to hydrogen’s low density, which creates upward buoyant force against the sealing layer. However, despite the lower density, H_2_ exhibits higher interfacial tension with brine compared to CH_4_ and CO_2_, resulting in a higher capillary entry pressure for H_2_, which improves the sealing effectiveness of the caprock.^51^ This can be assessed using the Bond number (*N*_B_), representing the ratio of buoyancy to capillary forces,^53–55^ i.e.,

$$N_{B}=\frac{\Delta \rho gd^{2}}{\sigma }$$ 3

where Δρ is the density difference between brine and gas, *g* is the gravitational acceleration, *d* is the representative length scale, and σ is the interfacial tension. The Bond number is very similar for hydrogen and methane (UHS and UGS),^56^ suggesting a similar risk of leakage through the caprock. However, besides interfacial tension, the contact angle also influences the capillary entry pressure, with lower contact angles leading to higher entry pressures. Unlike interfacial tension, it depends on fluid–fluid and fluid–solid interactions, including rock type and composition. This makes general comparisons complex, as caprock properties differ between systems and are therefore not included in this section. However, contact angles on shale, a common sealing rock type, are generally lowest for H_2_/brine, higher for CH_4_/brine, and highest for CO_2_/brine.^57^ Lower contact angles increase capillary entry pressure, indicating that the contact angle in UHS may slightly enhance the sealing potential compared to UGS and CCS.

As shown in Table 2, Bond numbers vary by less than 10% across UHS, UGS, and CCS, indicating a similar capillary sealing effectiveness. A similar conclusion was reached by Hashemi et al.^56^ for comparing H_2_ and CH_4_ contact angles.

In contrast to UGS and CCS, gas purity is a critical consideration in underground hydrogen storage. As a result, mixing between hydrogen and in situ or cushion gases becomes a major concern as it can significantly compromise hydrogen quality. Site-specific factors that influence gas mixing, such as reservoir heterogeneity and permeability contrasts, should therefore be carefully evaluated during the site selection process.

Furthermore, unlike natural gas in UGS, hydrogen will change the chemical equilibrium of the subsurface (reactive) environment. This reactivity can lead to geochemical interactions with minerals and formation fluids, potentially impacting both purity and storage efficiency.^7^ In addition, hydrogen is a universal energy carrier and acts as an electron donor for subsurface microbial communities, which may further affect gas composition and system performance through microbial consumption or transformation of hydrogen, as explained by Dopffel et al.^39^

## 4 Framework Setup

In this study, a framework is developed for the rapid site selection of Underground Hydrogen Storage in porous reservoirs. Building upon the work of Callas et al.,^16^ the framework consists of three stages. A summary of these stages is presented in Figure 1.

**Figure 1:**
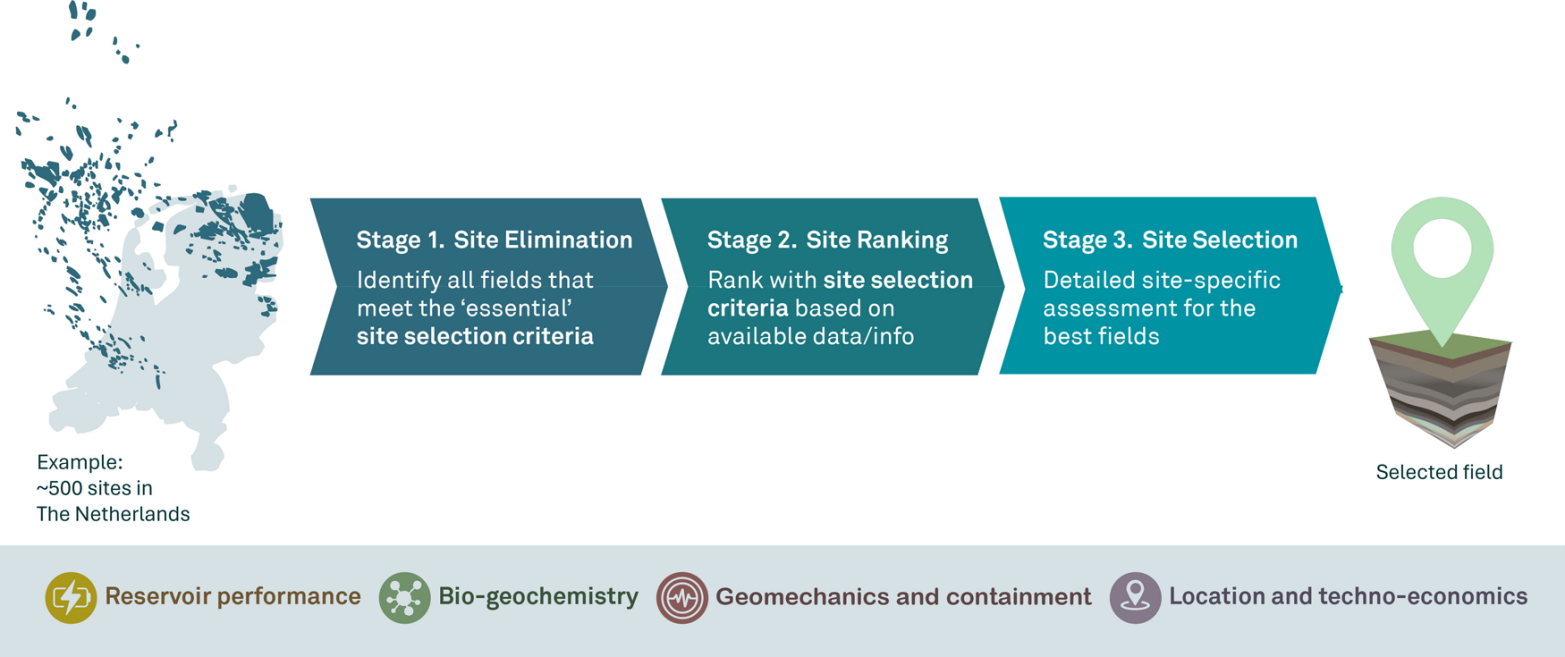
Overview of the multiscale framework for selecting optimal porous reservoirs for Underground Hydrogen Storage, inspired by Callas et al.^16^ The framework consists of three sequential stages: site elimination, site ranking, and site selection. At each stage, site selection is guided by criteria that are subdivided into four categories: Biogeochemistry, reservoir performance, Geomechanics and containment, and location and techno-economics. For illustration, the figure includes a map of The Netherlands showing its hydrocarbon fields (data with permission from NLOG.nl^64^), representing a typical group of reservoirs used for site selection.

In Stage 1: Site Elimination, sites are removed from consideration based on essential site selection criteria. These criteria consist of disqualifying thresholds, meaning that any site that fails to meet any of these thresholds is eliminated. This stage relies on readily available data.

In Stage 2: Site Ranking, the remaining sites are evaluated further. Sites are ranked according to a set of site selection criteria, some of which overlap with those from Stage 1. However, in this stage, each site is assigned a score based on these criteria, and a weight is applied to each factor.

Finally, in Stage 3: Site Selection, the top-ranked sites from Stage 2 are subjected to a detailed assessment based on dynamic simulations to determine their performance efficiency. However, in this study, we focus on Stages 1 and 2, as Stage 3 can vary significantly depending on the country and specific storage demands.

As explained previously, site selection criteria are used in Stages 1 and Stage 2. Underground hydrogen storage is a multidisciplinary field involving factors from various areas of expertise. Inspired by Callas et al.,^16^ we adapted the categorization of site selection criteria into four main groups: Bio-Geochemistry, Geomechanics and Containment, Location and Techno-Economics, and Reservoir Performance.

## 5 Reservoir Performance

Reservoir performance is a central consideration in the site selection for underground hydrogen storage. In this study, we define reservoir performance as the combination of geological characteristics, reservoir architecture, and operational conditions that influence the efficiency and feasibility of hydrogen storage. First, we introduce the key considerations and risks, followed by an assessment of how specific site selection criteria influence these factors.

### 5.1 Key Considerations and Risks

As in other subsurface gas storage applications, the injectivity and storage capacity are primary parameters. Injectivity, defined as the rate at which hydrogen can be injected per unit pressure drop,^21^ must be sufficient to meet operational demands without exceeding pressure constraints. Since UHS is a cyclic process, both injectivity and productivity should be considered. However, as the two terms describe similar concepts, they are used interchangeably in this paper.

Due to hydrogen’s unique thermophysical properties, specific risks such as gravity override, viscous fingering, segregation, and upconing in saline aquifers must be considered. These processes, along with gas mixing and residual trapping, can significantly affect the storage performance. Although solubility^7,65,66^ and diffusion^49,65,67,68^ effects are minor (typically below 1%) and thus excluded from this study, other transport-related phenomena remain central to the operational performance.

The objective of identifying optimal site selection parameters is to find reservoir characteristics that maximize injectivity while minimizing negative impacts from mixing, trapping, and flow instabilities, ultimately achieving the highest possible storage efficiency.

### 5.2 Reservoir Shape

Storage capacity or total pore volume is often named as a site selection criterion in literature,^12,25–28,41^ and even though it must align with project needs, large reservoirs might not be more efficient than smaller ones. In some cases, multiple smaller reservoirs may offer greater flexibility and efficiency than a single large one. Therefore, reservoir efficiency is often more important than absolute volume, and therefore, we do not classify this among the most influential parameters. However, storage capacity might be of influence for economic and infrastructure reasons, such as the working gas volume relative to the needed cushion gas volume and the availability of the infrastructure in the vicinity of the fields. This will be discussed further in Section 8.

Several simulation studies that performed sensitivity analysis showed that formation thickness plays a significant but context-dependent role in underground hydrogen storage. Thicker reservoirs generally provide greater pore volume, enabling higher hydrogen injection capacity and increased water displacement.^32^ However, thinner formations can offer advantages in terms of withdrawal efficiency and hydrogen purity due to shorter diffusion paths and steeper pressure gradients, particularly during early storage cycles. While thickness positively influences injectivity, its impact on recovery efficiency is more complex and depends on reservoir permeability, as shown in the sensitivity analysis of several studies.^26,31,36^ Therefore, there is often an optimal reservoir thickness rather than a general trend where increasing thickness consistently improves UHS. The optimal thickness depends on the reservoir characteristics and can be defined after further analysis, such as reservoir simulation. Therefore, thickness will not be taken into account as a site selection criterion since it requires further analysis, which is more suitable for stage 3.

Steeply dipping structures are widely regarded as favorable for underground hydrogen storage in both saline aquifers and depleted gas reservoirs, as demonstrated by various reservoir simulation studies.^26,31,38,69,70^ These structures consistently rank among the top site selection criteria due to their ability to reduce gas mixing, limit lateral hydrogen spreading, and enhance gravitational segregation. In saline aquifers, steep dips also help to mitigate water upconing during withdrawal, thereby improving recovery efficiency and containment.^48,69^ Overall, a positive correlation between the dip angle and storage performance has been observed. Even though a minimum dip angle is not included in the screening stage of other studies,^26,31^ we recommend avoiding flat reservoirs with dips less than 5 degrees, as the very low density of H_2_ will cause the plume to spread laterally over a large distance, making it hard to recover back. Structures with higher dip angles provide greater benefits compared to those with lower dips, which is reflected in the ranking phase.

### 5.3 Operational Conditions

Depth, pressure, and temperature are widely recognized as key criteria for underground hydrogen storage site selection (Table 1). While these parameters are interrelated, pressure becomes a more reliable indicator in depleted gas reservoirs, where prior production decouples it from depth.

Identifying optimal conditions is a complex task, as these properties affect the thermophysical properties, injectivity, recovery efficiency, microbiological activity, compression costs, and risk of caprock leakage. This section focuses specifically on their influence on reservoir performance, defined here as injectivity and recovery efficiency. Among these, sensitivity analyses show that pressure consistently emerges as the most critical factor, while temperature plays a relatively minor role.^26,31,71^ Therefore, the temperature is excluded from the most influential site selection criteria for Reservoir Performance proposed in this study.

The thermophysical properties of hydrogen vary with depth due to the increasing pressure and temperature. Assuming a linear geothermal gradient of 33 °C/km and a hydrostatic pressure gradient of 10 bar/100 m, the density of H_2_ nearly doubles between depths of 1000 and 2000 m, while viscosity increases by only about 10%. As a result, the volumetric energy density increases significantly more than the viscosity, leading to a theoretical improvement in productivity with increasing depth.

Therefore, some studies advocate for deeper reservoirs with higher pressures to achieve improved volumetric energy density.^41,51^ For example, Chen et al.^36^ found in their sensitivity analysis that greater depth and pressure positively influence hydrogen recovery efficiency and injectivity, though their model does not account for the effect on roundtrip efficiency due to an increase in compression energy needed. Similarly, Buscheck et al.^72^ suggested deeper reservoirs with greater pressure because it maximizes the volumetric energy storage density. Besides that, they found that the effect of viscous fingering and gravity override will be decreased at higher pressures.

It is worth highlighting that in contrast to CO_2_, hydrogen density increases smoothly with increasing depth. As such, the gain in the amount of energy storage at deeper reservoirs is much less relevant for UHS than for CO_2_ storage. Indeed, CO_2_ turns supercritical at depths higher than ∼800 m; as a result, its density jumps significantly to higher values. This is not the case for hydrogen.^73^ Indeed, Okoroafor et al.^26^ and Sekar et al.^31^ show with a simulation study that productivity decreases with increasing reservoir pressure (and higher depths) due to higher compression energy demands. Consequently, lower pressures, typically found in shallower reservoirs, are considered to be more favorable, and these studies recommend assigning higher scores to low-pressure sites.

Other studies focus on balancing depth with caprock sealing effectiveness by performing a more theoretical analytical modeling study to identify the optimal storage depth. They propose optimal depths of 1100–1600 m to maximize hydrogen capacity while minimizing leakage risk.^74,75^

Regarding depth limits, Okoroafor et al.^26^ recommend excluding fields deeper than 3000 m, citing declining productivity. This is consistent with other studies, which also support depth limits in this range for decreased storage volume while avoiding exceeding the capillary breakthrough.^74,75^ Finally, to ensure withdrawal without artificial lift, it is proposed that reservoir pressure must exceed wellhead pressure by at least 1 bar per 100 m of depth, accounting for gravity and friction losses in the wellbore.^26^

Effects of depth, pressure, and temperature on other aspects of site selection such as biogeochemistry, economics, and geomechanics will be discussed in the next sections.

### 5.4 Rock Properties

Although high porosity enhances storage capacity, it is not considered a key factor influencing injectivity or hydrogen recovery efficiency based on sensitivity analysis of reservoir simulation studies.^26,31,36^ In contrast, permeability is consistently identified as one of the most influential reservoir properties for UHS performance.^26,32,36^ Generally, higher permeability improves hydrogen recovery and injectivity;^26,31^ however, in saline aquifers, very high permeability can slightly reduce recovery efficiency due to increased water production from upconing.^36^ To ensure adequate performance, permeability and porosity are indeed required to be higher than some minimum values. The literature suggests the minimum values of 50 mD and 10% for permeability and porosity, respectively.^26^

Reservoir heterogeneity is another key factor influencing the UHS performance. Homogeneous systems enhance both productivity and storage efficiency by supporting more uniform gas flow and reducing phase interference.^26,38,76^ In contrast, high-permeability layers may lead to excessive lateral hydrogen migration, while low-permeability barriers can hinder upward gas flow during withdrawal, promoting increased water production.^26,48^ In scenarios involving cushion gases or residual hydrocarbons, reservoir homogeneity becomes even more important: it helps to minimize mechanical dispersion and gas mixing, improving hydrogen purity in the production stream.^38,76^ This can potentially mean that the expected performance in fairly homogeneous sandstones is higher than that in heterogeneous fractured carbonates.

In contrast to the oversimplified definitions of heterogeneity in the UHS literature either by assuming layered-permeability distributions^26^ or using hard-to-quantify depositional environment,^38^ we propose to use the Dykstra–Parsons coefficient^77^ to describe the degree of heterogeneity, i.e.,

$$V=\frac{K_{50}-K_{84.1}}{K_{50}}$$ 4

Here, *V* is the Dykstra–Parsons coefficient, *K*_50_ is the median permeability, and *K*_84.1_ is the permeability at the 84.1th percentile. The coefficient quantifies the degree of permeability variation; values close to 0 indicate a homogeneous reservoir, while values approaching 1 signify high heterogeneity.

Finally, while a low anisotropy ratio (*k*_v_/*k*_h_) is generally preferred to support vertical hydrogen migration, so far, it has not yet been found to be a highly sensitive parameter.^26^

## 6 Bio-Geochemistry

The injection of hydrogen into porous geological reservoirs can alter the chemical equilibrium among formation water, dissolved gases, and the rock matrix. This disturbance may initiate a variety of geochemical reactions.^7^ Additionally, hydrogen serves as an electron donor for a wide range of microbial processes in the subsurface.^39,40^ These biogeochemical processes can pose several risks, such as hydrogen consumption and contamination, biofilm formation, clogging of flow pathways, mineral dissolution or precipitation impacting injectivity, the formation of leakage pathways, and the degradation of caprock integrity.^7,39,78^

Biogeochemical reactions are typically classified into biotic (microbial) and abiotic (geochemical) processes. While recent studies suggest that geochemical processes alone may not pose a significant risk to the feasibility of underground hydrogen storage in porous reservoirs,^31,66,79^ a strong interaction between biotic and abiotic reactions has been observed.^80,81^

The diversity of microbial species combined with the wide variety of possible reaction pathways and the varying environmental conditions that favor different microbial groups makes it extremely difficult to predict the extent and impact of subsurface hydrogen reactions. Nonetheless, it is evident that biogeochemical processes can substantially affect the efficiency and safety of UHS operations^39,78^ and must therefore be carefully evaluated during site selection and project design.

Multiple factors influence the likelihood and severity of biogeochemical risks. Based on the literature,^7,31,39,40,68,78,82^ the most critical parameters for microbial activity are temperature, pH, and salinity. Microbial growth is likely within temperature ranges of 20–80 °C, pH values between 3 and 8, and salinities below 100 g/L, as explained by Dopffel et al.^39^ Therefore, within this range, microbial risks should be assessed site-specifically. However, microbial activity cannot be ruled out, even outside these ranges. Additional important parameters include brine composition and rock mineralogy, which will be further discussed in the following subsections.

### 6.1 Temperature

Temperature plays a crucial role in determining microbial viability and activity.^7,39,40,78^ According to the microbial risk classification proposed by Thaysen et al.,^78^ the upper temperature limit for microbial life is approximately 122 °C,^83,84^ beyond which microbial processes are not expected. A low microbial risk is associated with temperatures above 90 °C. A medium risk is identified for the range between 55 and 80 °C, depending on reservoir salinity. Below 55 °C, microbial activity is considered high-risk due to optimal growth conditions for many hydrogen-consuming microbial species, including species with a high salinity tolerance.

### 6.2 Brine Composition

Together with the temperature, the pH and salinity of the formation water are key controls on microbial activity. Microorganisms commonly found in subsurface environments, such as methanogens, sulfate-reducers, homoacetogens, and iron­(III) reducing bacteria—typically exhibit optimal growth at pH values between 6.0 and 7.5.^7,40^ However, active microbial processes have also been reported in a broader pH range of 3–8,^39^ with reduced activity expected outside this range.

Higher salinities are generally beneficial for minimizing microbial risks. Above 100 g/L, microbial diversity and activity are significantly reduced.^39^ In reservoirs with salinities above 100 g/L and temperatures exceeding 55 °C, no cultivated hydrogen-consuming microbes have been identified.^78^ However, at lower temperatures, certain microbial species may tolerate extreme salinity conditions.

For site-specific assessments, other key factors include the presence of electron acceptors (e.g., sulfate, nitrate, and ferric iron), carbon sources (e.g., carbonate/CO_2_, organic compounds), and the brine activity coefficient. Measurements of total or metabolically active microbial cell counts, as well as microbial community analyses, are valuable tools for evaluating microbial risks.^39,40,78^

### 6.3 Rock Composition

The composition and mineralogy of the reservoir rock significantly influence both biotic and abiotic reactions. Sandstone reservoirs with high quartz content and low concentrations of reactive minerals such as calcite, carbonates, sulfates (e.g., anhydrite), and clays are generally preferred to minimize reaction potential.^7,31,39,45,81,82,85^ In such lithologies, hydrogen losses due to abiotic reactions have been reported to be negligible.^86^ In contrast, formations containing higher amounts of reactive minerals, particularly carbonate rocks such as limestone and dolomite, have been associated with more significant geochemical reactions, resulting into significant H_2_ loss of up to 9.5% and porosity reduction of up to 47%.^82,87,88^ It is worthwhile to highlight that the extent of these reactions varies widely depending on the experimental conditions and rock chemical compositions; notably, some studies have reported only minor exposure effects for carbonate rocks.^89,90^

## 7 Geomechanical Risks and Containment

The cyclic injection and withdrawal of hydrogen in underground reservoirs cause repeated pressure fluctuations, directly impacting the subsurface stress regime. These variations can induce rock deformation, alter porosity and permeability, reduce caprock sealing efficiency, and lead to induced seismicity via fault reactivation, subsidence or uplift, and wellbore instability.^7,91,92^ As such, geomechanical integrity is critical to ensuring both containment and operational safety in underground hydrogen storage.

Depleted gas reservoirs are advantageous over aquifers due to their proven containment performance and better-characterized mechanical behavior. However, their suitability still depends on local stress conditions, fault activity, and caprock integrity. For a comprehensive review on geomechanical considerations and site selection guidance, see Kumar et al.^91^

Biogeochemical processes can further influence geomechanical stability by altering pore pressure, reducing mineral cohesion, and changing rock permeability, thereby contributing to stress redistribution, fault weakening, and increased risk of deformation or leakage.^91^ Wellbore instability should be considered during the implementation of UHS, e.g., by selecting the right materials and implementation of suitable monitoring methods to detect leakage of H_2_ along the wellbore.^91^ In addition to the operational wells, abandoned wells can also pose a risk for hydrogen leakage, so the presence, condition, and age of pre-existing wells should be carefully evaluated as part of the site-specific selection process (stage 3).^34^

One of the most effective ways to mitigate geomechanical hazards, such as seismicity, subsidence, and leakage through the caprock, is to select a site with a well-characterized geomechanical profile, providing insight into rock properties, in situ stress, and failure mechanisms.^91^ However, full geomechanical characterization is time-consuming and may not be feasible during the early screening phases. Therefore, it is recommended that detailed geomechanical analyses be reserved for the final selection stage and applied only to the most promising sites.

Geomechanical risks can be broadly categorized into two main groups: leakage through the caprock and seismicity due to fault reactivation. These are discussed along with potential site selection criteria in the following sections with a focus on efficient site screening using readily available data.

### 7.1 Caprock

Kumar et al.^91^ emphasize the importance of thick, low-permeability caprocks, consistent with other proposed site selection frameworks.^26,41,42,93^ In stage 1 of site screening, depleted gas fields can be assumed to have adequate sealing capacity due to their containment history (see Section 3 for a comparison between methane and hydrogen). For aquifers, minimum caprock thicknesses of 10–50 m and a minimum permeability of 0.01 mD are suggested in literature.^26,30,41,42,93^ However, these numbers are generally not based on numerical simulations or experimental measurements. In our study, we propose a minimum caprock thickness of 30 m, based on the pore network study of Wang et al.^94^ They concluded that with lower thicknesses leakage becomes significant. Their study included multiple shale samples, of which the maximum permeability is 0.02 mD, on which we base our minimum permeability value on. In stage 2, sites with thicker and less permeable caprocks are preferred, with depleted fields scoring higher than those of aquifers.

As discussed in Section 5, the column height of safely stored hydrogen depends on pressure, temperature, and depth, which, in turn, affects the risk of capillary breakthrough into the caprock. Optimal storage depths between 1100 and 1600 m and a maximum around 3000 m are recommended.^74,75^

Excessive injection pressure can fracture the caprock, creating potential leakage pathways. Sekar et al.^95^ propose evaluating sites based on the ratio of injection pressure to fracture pressure, the latter being calculable from in situ stresses, Biot’s coefficient, Poisson’s ratio, pore pressure, and tensile strength. While this is a valuable analysis, we recommend it as part of the detailed site evaluation in stage 3.

Caprock type and composition are critical to seal integrity. As discussed earlier, wettability and pore size control capillary entry pressure, with small-pore, water-wet systems being most favorable.^96^ Shales, salt rocks, and anhydrites are commonly considered caprocks due to their low permeability and small pore sizes. Salt typically has the lowest permeability and pore size, resulting in very high capillary entry pressures.^97^ Shale is the most frequently mentioned caprock in the literature and provides effective sealing due to its microporous structure and high tortuosity.^97^ Anhydrites have slightly larger but isolated pores, which still lead to sufficiently low permeability.^97^ Caprocks containing reactive minerals, such as carbonates or sulfates, are generally less ideal, as geochemical reactions may compromise long-term integrity^7,98^ (see Section 6). Therefore, careful evaluation of the caprock composition is essential.

In conclusion, thickness and permeability are the most influential and easily screenable parameters, which are therefore considered in stages 1 and 2. However, other parameters such as caprock type, pore structure, wettability, composition, and mechanical properties should be taken into account in the third stage.

### 7.2 Faults and Seismicity

Faults present a dual risk in UHS: they can act as potential leakage pathways and may also be reactivated under cyclic pressure changes, potentially compromising caprock integrity and triggering induced seismicity.^91^ Therefore, sites with extensive faulting should be avoided.^16,26^ However, in specific cases, faults can also be beneficial if they are sealing, as they may act as structural traps. If faults are present in shortlisted sites, a detailed geomechanical analysis should be conducted during stage 3 to assess their stability and sealing potential.

Sites near active faults or those that have experienced recent seismic events should be naturally eliminated early in the screening process, as pressure build-up may trigger fault reactivation.^16,26^ In addition, we recommend performing baseline monitoring surveys to quantify sensitive weak zones as well as the current level of activities before any operation starts.

## 8 Location and Techno-Economics

Location and techno-economic considerations are not always directly considered in site selection studies on UHS. However, in real-world project development, these factors often become the primary drivers of site selection and overall feasibility. In the following sections, we focus on the key factors that influence the techno-economic performance and location of potential UHS sites.

### 8.1 Techno-Economics

The techno-economics of Underground Hydrogen Storage are primarily governed by capital expenditures (CAPEX), such as drilling and cushion gas injection, and operational expenditures (OPEX), including monitoring and maintenance. The levelized cost of storage (LCOS) integrates these costs over the project’s lifetime to evaluate the total costs per stored unit of H_2_.

Depleted reservoirs are typically favored over aquifers due to the presence of existing infrastructure and residual gas which lowers the demand for cushion gas, which lowers both CAPEX and the need for extensive site characterization.^99,100^ The LCOS is also found to be lower for depleted gas reservoirs.^101^

Cushion gas is typically the largest contributor to LCOS, often exceeding 50% of total costs.^33,100–102^ Selecting reservoirs with a high working gas to cushion gas (WG/CG) ratio can therefore significantly reduce costs.^28^ Other major cost drivers include compression and gas cleaning, whereas wells and piping contribute relatively little (3–5%) according to.^102^ However, this study does not consider leakage from the piping. Baghirov et al.^103^ found that the transportation of H_2_ contributes 84% to the CO_2_ equivalent emissions of green UHS projects. This was the case due to leakage from the pipelines. Therefore, we do consider the distance to any H_2_ network as a factor in our ranking phase.

A common assumption is that depleted gas reservoirs require approximately 50% cushion gas (a WG/CG ratio of 1:1), while aquifers require up to 80% (a WG/CG ratio of 1:4).^104–106^ However, actual ratios vary widely depending on reservoir properties.^107^ Site selection should therefore aim to minimize cushion gas requirements, but precise estimation of absolute volumes requires more data and can be time-consuming. Therefore, this is more appropriate for detailed feasibility studies. During the initial screening phase (Stage 2), if the WG/CG ratio is not known, we propose to assume 1 for depleted gas fields and 0.25 for aquifers and to focus on more general geological criteria known to influence cushion gas demand.

Heinemann et al.^107^ identified reservoir permeability, depth, and trap geometry as key controls on the WG/CG ratio. Zhao et al.^33^ also found that reservoirs with higher porosity and permeability are more cost-effective for hydrogen storage, primarily due to lower cushion gas requirements. High permeability improves pressure dissipation, enabling greater working gas injection with less cushion gas. Greater depth provides a wider operational pressure window, which also reduces the cushion gas needs. However, deeper reservoirs also require more compression electricity, which is also a significant part of the levelized costs.^26^ Trap geometry may influence brine displacement and injection performance; lower dip angles reduce pressure buildup during injection, improving the WG/CG ratios. However, in depleted gas fields where brine saturation is lower, the effect of the dip angle is expected to be less significant. Conversely, steeper dips may improve hydrogen recovery by enhancing buoyancy-driven upward migration, as discussed in Section 5. The effect of the total reservoir capacity on the LCOS is expected to be minimal.^101^

In conclusion, minimizing the WG/CG ratio is essential for reducing LCOS. This is best achieved in deeper, highly permeable reservoirs, which also offer advantages for injectivity, productivity, and geomechanical stability.

### 8.2 Location

In addition to the economic considerations discussed in the previous section, several spatial and regulatory factors must also be taken into account during site selection. Certain fields must be excluded from consideration if they fall within zones reserved for other purposes, such as wind farms, shipping lanes, military operations, or environmentally protected areas. These constraints significantly reduce the number of viable locations for Underground Hydrogen Storage. Figure 2 presents a map of the hydrocarbon fields in the North Sea, in combination with other uses of the North Sea, illustrating the extent to which spatial constraints limit the availability of suitable sites.

**Figure 2:**
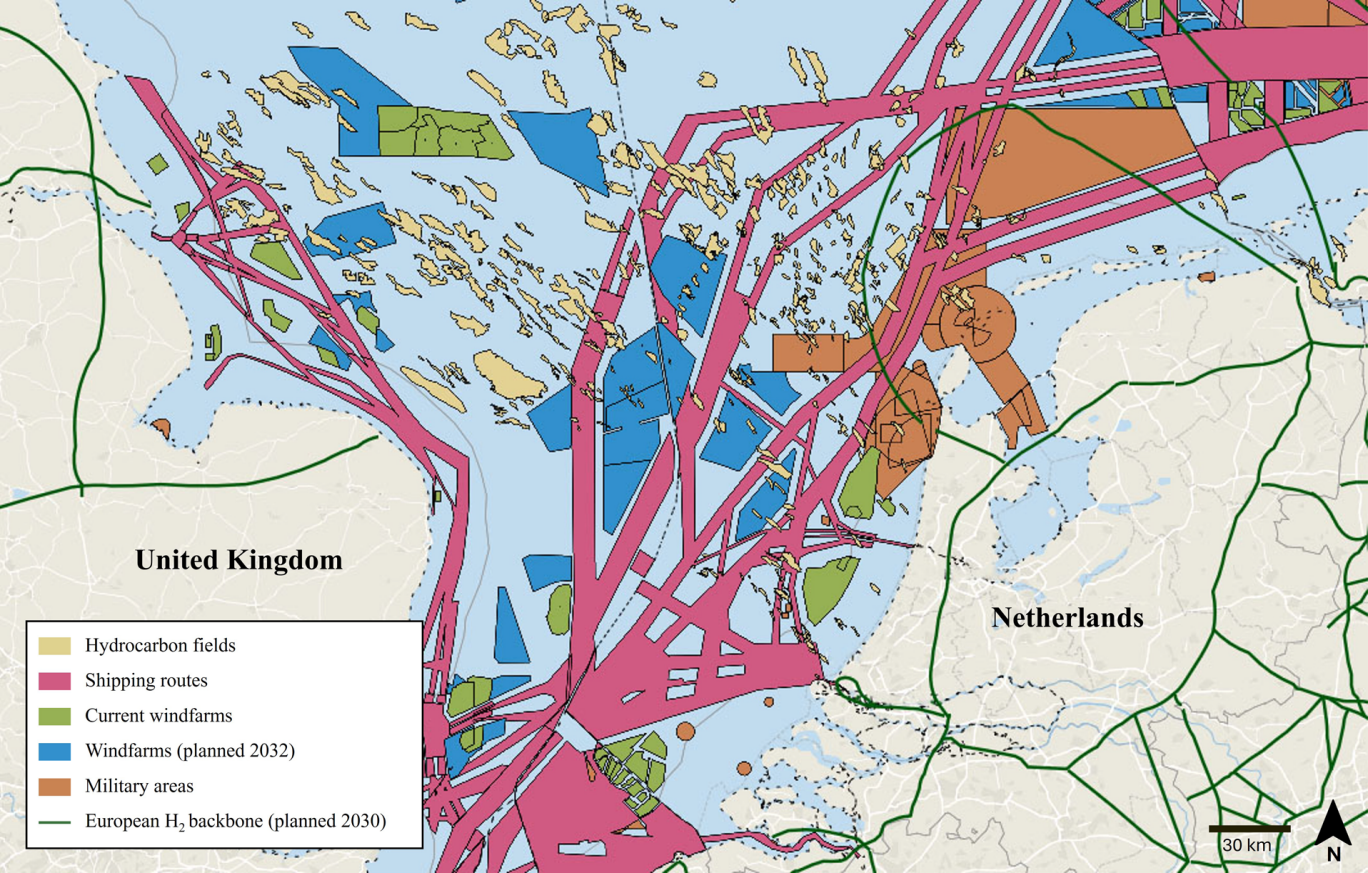
Map of the North Sea showing offshore hydrocarbon fields and key spatial constraints relevant to site selection, with permission from the North Sea Energy Atlas (https://north-sea-energy.eu/atlas).^108^

Furthermore, public perception and the perceived risks associated with hydrogen storage, particularly for onshore sites, can influence the feasibility of a given location. In areas with high population density, these concerns are often more pronounced and may impact regulatory approval and project acceptance. While these societal factors play a crucial role in later project stages (as discussed in Section 10), they are highly context specific and difficult to generalize and quantify. We therefore choose not to include them in the initial screening and instead focus primarily on excluding fields located within restricted or protected zones and their distance to a H_2_ network.

## 9 Framework

Based on the combined analysis of the previous sections, a multidisciplinary systematic framework is developed, which focuses on the most influential and practically applicable criteria to enable effective field elimination and ranking. The criteria for both elimination and ranking are summarized in Figure 3. As described in the following sections, many criteria might overlap among different categories. The criteria are displayed under the category that is most suitable.

**Figure 3:**
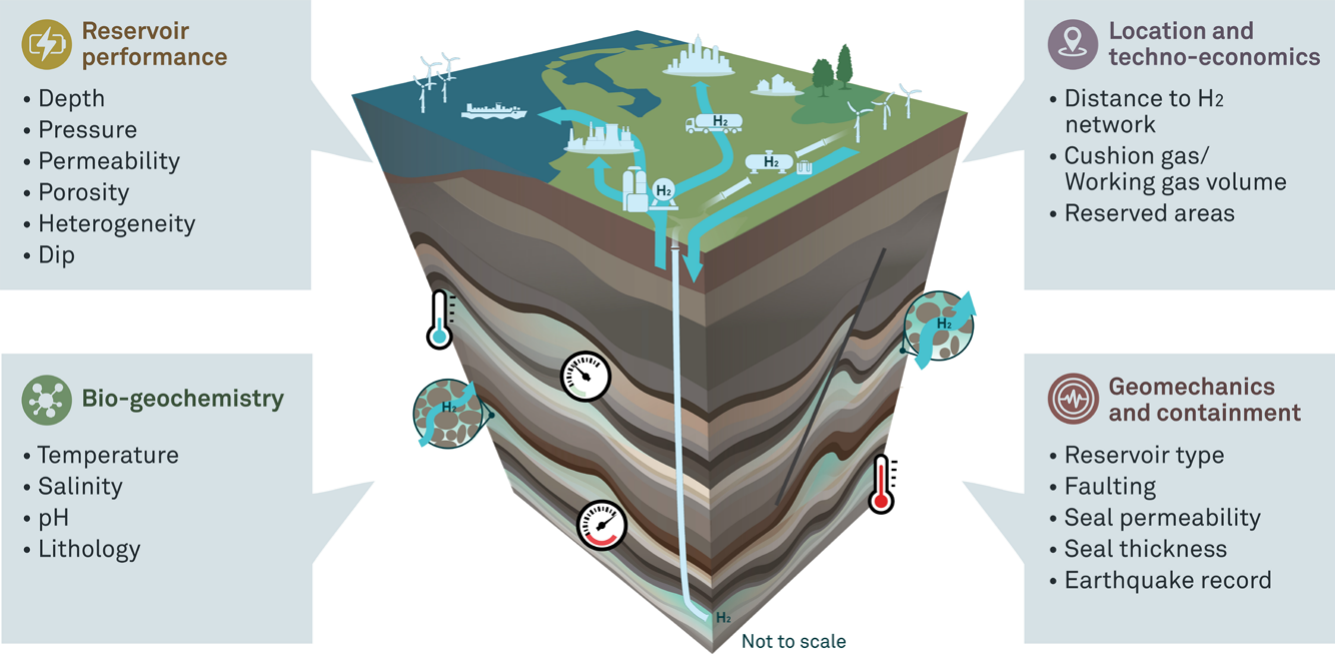
Most important site selection criteria for the efficient ranking and elimination of potential fields for UHS.

The selection criteria are subdivided into elimination criteria and ranking criteria, which are quantified in the following sections. These criteria are, as much as possible, evidence-based—derived from experimental studies, numerical simulations, or well-established reasoning. However, quantifying precise values remains challenging, as they often depend on site-specific conditions and multiple interacting factors. As a result, we rely on general trends that indicate whether a parameter is likely to have a positive or negative impact on the suitability of a site for Underground Hydrogen Storage. The methodology used to derive these values is described in detail within each respective category. The sources informing our criteria are listed in the subsequent tables, though in some instances, we determined the final numerical values ourselves, based on a synthesis of multiple referenced studies.

### 9.1 Stage 1: Elimination

Table 3 presents the elimination criteria proposed in this study. Any field exhibiting one or more properties that fall within these thresholds should be excluded from consideration for Underground Hydrogen Storage, based on our proposed framework.

**Table 3:** Disqualifying Thresholds Used in Stage 1: Site Elimination Is to Eliminate Sites That Do Not Meet Essential Criteria^a^

criterion	disqualifying condition
Reservoir Performance	
depth	>3000 m^26,74,75^
pressure	<wellhead pressure + (0.01 bar/m) × reservoir depth^26^
permeability	<50 mD^26^
porosity	<10% ^26,42^
net reservoir thickness	<10 m^26,42^
reservoir dip	<5 deg
Geomechanical Risks and Containment	
distance to active faults	<4 km^109^
earthquake record	<5 km magnitude 5 (from 2015 to present), <10 km magnitude 5 (from 1769 to present)^26,109^
top seal thickness^b^	>30 m^94^
Top seal permeability*	>0.02 mD^94^
Location and Techno-Economics	
policies	protected areas, reserved areas for e.g., oil/gas, shipping routes, windfarms, military activities

a If a field meets any of the listed conditions, it is excluded from further consideration.

b Only applicable when top seal integrity is unproven, such as aquifers without prior gas containment.

### 9.2 Stage 2: Ranking

Table 4 summarizes the most influential ranking criteria identified in this study. The ideal field characteristics are listed in the rightmost column, while the least favorable properties are shown in the second column from the left.

**Table 4:** Evaluation Criteria for Stage 2: Site Ranking

criteria	1 (worst)	2	3 (best)
Reservoir Performance			
depth [m] ^26,31,36,41,51,74,75^	<500; >2000	500–1000; 1500–2000	1000–1500
pressure [bar] ^26,31,36,41,51,74,75^	<50; >200	50–100; 150–200	100–150
permeability [mD] ^26,32,36^	<500	500–1000	>1000
permeability heterogeneity *V* ^26,38^	0.8–1	0.5–0.8	0–0.5
dip [deg] ^26,38,70,110^	5–10	10–15	>15
Purity: Bio-Geochemistry			
salinity [g/L] ^39,40,82^	<50	50–100	>100
temperature [°C] ^7,39,40^	40–60	60–80	>80
pH^7,39,40^	6–7.5	3–7.5 or 7.5–8	<3 or >8
lithology^31,39,45,81,82,85,86^	carbonate		sandstone (pure)
Geomechanical Risks and Containment			
type of reservoir	aquifer		depleted gas field
faulting^16^	extensively faulted	moderately faulted	limited faulted
seal permeability [mD] ^26,41,42,93^	0.005–0.01	0.001–0.005	<0.001
seal thickness [m] ^91,93^	20–40	40–100	>100
Location and Techno-Economics			
distance to H_2_ network	>60 km	30–60 km	<30 km
working gas/cushion gas ratio^a^ ^28^	<0.25	0.25–1	>1

a If the absolute value of this ratio is not known, a value of 0.25 can be assumed for aquifers and a value of 1 can be assumed for depleted gas fields.

Depth and pressure are critical parameters influencing injectivity, recovery efficiency, gas purity, and cost in UHS. Higher depths generally lead to increased pressure, which improves the volumetric energy density, reduces viscous fingering, and enhances hydrogen containment. However, deeper reservoirs require more compression energy, which increases operational costs and may reduce the round-trip efficiency. Additionally, deeper reservoirs are harder to monitor, which is an important factor, especially for pilot studies.

Pressure, more than depth, has been identified in several sensitivity analyses as the most influential operational parameter. Higher pressures can improve injectivity and reduce mixing but may lower net energy efficiency due to compression requirements. Additionally, while the storage density increases at greater depths, the risk for capillary leakage also increases at a deeper depth.

Taking this all into account, we suggest that intermediate depths are optimal for UHS, balancing the effects of various processes. Therefore, we suggest optimal depths between 1000 and 1500 m and corresponding pressures of 100–150 bar (assuming hydrostatic pressures), which is in the same range as proposed in the literature.^74,75^

## 10 Challenges and Perspectives

Recently, substantial knowledge of underground hydrogen storage (UHS) has been gained. Significant advances have been made across the various disciplines involved, and when combined with existing experience from the oil and gas industry, carbon capture and storage (CCS), and underground gas storage (UGS), they provide a strong basis to derive the most influential screening and elimination criteria for UHS, as presented in Section 9.

This framework is developed to be applicable to any group of reservoirs. For example, in The Netherlands, there are over 500 depleted oil and gas fields.^64^ These fields exhibit diverse characteristics, including large variations in depth, pressure, geometry, fluid properties, and more.^64^ Identifying suitable fields among these options requires careful balancing of these different parameters. Applying the framework to such a data set enables systematic screening and ranking, helping to select the most promising fields for further site-specific evaluation and final selection.

However, UHS in porous media still presents several unresolved challenges relevant to site selection that require multidisciplinary research. A key issue is the uncertainty regarding the relative importance of specific site selection criteria. For example, the importance of the biogeochemical category in comparison to other criteria. Due to the limited number of operational projects, there is insufficient practical evidence on how microbial activity and chemical reactions in the subsurface may impact hydrogen purity and storage performance in real fields. Pilot-scale projects are therefore essential to validate current assumptions and refine selection frameworks. The results of pilot-scale projects are important future research direction.

Recently, natural hydrogen extraction has gained increasing attention.^111–113^ Numerous natural H_2_ sources have been documented globally.^113^ In the current absence of extensive practical experience with UHS, these natural hydrogen systems could serve as valuable ”living laboratories”. They offer the opportunity to study hydrogen behavior in the subsurface and may provide key insights that are currently lacking. For example, regarding the variety of structural traps in which H_2_ can be safely stored.^113^

In terms of economic feasibility, large uncertainties remain due to the early stage development of the hydrogen market. For example, the future price and availability of hydrogen, as well as the demand for hydrogen, will play a large role in the economics of UHS projects and consequently in the site selection.

Societal embeddedness is another critical challenge. Experiences from onshore CO_2_ storage in Europe, e.g., the canceled Barendrecht project in The Netherlands,^114^ have shown that technically sound projects may fail without public support. Although societal factors are not included in this framework, because they are highly region-specific and difficult to quantify in a generalized way, they should be addressed explicitly in the later stages of site selection. As such, public perception and local regulatory responses must be taken into account during Stage 3 and all subsequent stages of project development.^115,116^

Some important technical aspects, such as reservoir thickness, caprock composition and integrity, abandoned wells, and fault activation, are suggested to be taken into account in the last stage of site selection (Stage 3), as they are highly case-dependent, and essential data is not always easily accessible. However, these factors do define the suitability of a site. Therefore, future work should focus on describing in detail how such criteria can be evaluated in Stage 3, and how a final decision for the most suitable field can be made.

## 11 Conclusions

This study critically assesses existing literature and domain-specific studies spanning multiple disciplines relevant to Underground Hydrogen Storage (UHS) to identify the most influential and practically assessable criteria for site selection. Drawing from this evaluation, we present a systematic framework for selecting UHS sites in porous media, integrating considerations from reservoir performance, geomechanics and containment, biogeochemistry, and techno-economics. The resulting methodology enables the efficient and reliable elimination and ranking of a large number of candidate fields. It consists of 11 site elimination criteria and 15 site screening criteria for early stage decision-making in a scientifically grounded and efficient manner. While detailed site-specific assessments remain essential in later phases, this framework provides a robust foundation for consistent and transparent initial evaluations.

We recommend following the technical procedures presented in this review to screen all potential sites for UHS in depleted reservoirs and identify the most suited. However, as also identified in the IEA Technology Collaborative Program 42 on UHS reports,^115,116^ it is crucially important to highlight the societal embeddedness and the availability of the infrastructure for final site selection strategies.
